# Pseudopeptide Amyloid Aggregation Inhibitors: In Silico, Single Molecule and Cell Viability Studies

**DOI:** 10.3390/ijms22031051

**Published:** 2021-01-21

**Authors:** Morgan Robinson, Jennifer Lou, Banafsheh Mehrazma, Arvi Rauk, Michael Beazely, Zoya Leonenko

**Affiliations:** 1 Department of Biology, University of Waterloo, Waterloo, ON N2L 3G1, Canada; m9robinson@uwaterloo.ca; 2 School of Pharmacy, University of Waterloo, Waterloo, ON N2L 3G1, Canada; 3 Department of Physics and Astronomy, University of Waterloo, Waterloo, ON N2L 3G1, Canada; jenniferwulou@gmail.com; 4 Department of Chemistry, University of Calgary, Calgary, AB T2N 1N4, Canada; bmehrazm@ucalgary.ca (B.M.); rauk@ucalgary.ca (A.R.)

**Keywords:** Alzheimer’s disease, amyloid-β, aggregation inhibitors, neuroprotection, HT22 cells, molecular dynamics, atomic force microscope

## Abstract

Neurodegeneration in Alzheimer’s disease (AD) is defined by pathology featuring amyloid-β (Aβ) deposition in the brain. Aβ monomers themselves are generally considered to be nontoxic, but misfold into β-sheets and aggregate to form neurotoxic oligomers. One suggested strategy to treat AD is to prevent the formation of toxic oligomers. The SG inhibitors are a class of pseudopeptides designed and optimized using molecular dynamics (MD) simulations for affinity to Aβ and experimentally validated for their ability to inhibit amyloid-amyloid binding using single molecule force spectroscopy (SMFS). In this work, we provide a review of our previous MD and SMFS studies of these inhibitors and present new cell viability studies that demonstrate their neuroprotective effects against Aβ(1–42) oligomers using mouse hippocampal-derived HT22 cells. Two of the tested SG inhibitors, predicted to bind Aβ in anti-parallel orientation, demonstrated neuroprotection against Aβ(1–42). A third inhibitor, predicted to bind parallel to Aβ, was not neuroprotective. Myristoylation of SG inhibitors, intended to enhance delivery across the blood-brain barrier (BBB), resulted in cytotoxicity. This is the first use of HT22 cells for the study of peptide aggregation inhibitors. Overall, this work will inform the future development of peptide aggregation inhibitors against Aβ toxicity.

## 1. Introduction

### 1.1. Amyloid-β Cascade as A Target for Alzheimer’s Disease Treatment

Alzheimer’s disease (AD) is a neurodegenerative disease characterized by the accumulation of toxic, misfolded, and aggregated amyloid-β (Aβ). The Aβ monomer is a 39 to 43 residue peptide fragment produced endogenously within neurons from the cleavage of the transmembrane amyloid precursor protein (APP) by two secretase complexes: β- and γ-secretase [1]. An imbalance between Aβ production and clearance in brains of individuals with AD results in increased levels of toxic aggregates [2,3]. It is now widely recognized that soluble Aβ oligomers exhibit the greatest neurotoxicity as compared to the monomer and fibril states of the protein [4,5,6]. Thus, preventing oligomerization may be a viable strategy for mitigating Aβ toxicity in AD [7].

Peptide-based Aβ aggregation inhibitors are potential preventative strategies that have some advantages as compared to monoclonal antibodies (mAbs), including low immunological profile, small size, and tunable, drug-like characteristics. To date, anti-Aβ monoclonal antibodies (mAbs) have been the focus of clinical trials that target Aβ pathology in AD. Unfortunately, these clinical trials have not lived up to the expectations suggested by preclinical studies in AD animal models, leading many to doubt the prevailing amyloid cascade hypothesis [8,9,10]. Early clinical evidence suggested that the mAb, Solanezumab, may change disease trajectory when administered early or in pre-clinical AD stages, but unfortunately, phase 3 trials were not successful [11,12]. Other antibodies such as aducanumab have demonstrated the ability to reduce Aβ deposition in early and pre-AD patients [13], however the effects on cognitive decline in AD patients are less clear.

**Figure 1:**
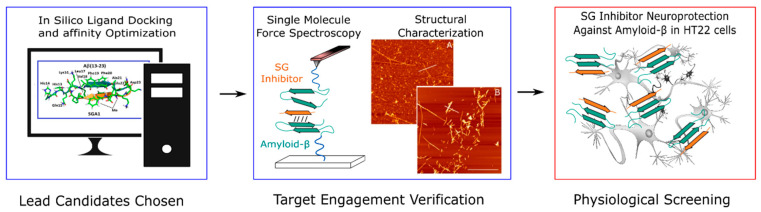
Drug Development Pipeline. In the first part, Aβ in silico screening of an SG inhibitor library is performed, wherein the inhibitors with optimal binding affinities were selected for further characterization. Select inhibitors chosen from affinity optimization were screened in single molecule force spectroscopy (SMFS) studies to verify target engagement and ability to reduce Aβ-Aβ binding (blue, these studies were previously reported). Inhibitors were then screened for toxicity and neuroprotection against Aβ, results presented for the first time in this article (orange).

Peptide Aβ aggregation inhibitors have been shown to reduce Aβ aggregation kinetics and modify the structure of Aβ aggregates prepared in vitro by preventing fibrillization [14,15,16,17,18,19,20,21]. Several of these Aβ peptide inhibitors have demonstrated positive anti-neurotoxic effects against Aβ in vitro; in addition, reductions in pathological markers, including Aβ deposition, fibrillogenesis and oxidative stress, as well as improvements in memory, have been observed in vivo [14,17,22,23,24,25,26]. The potential of Aβ targeted therapeutics as a preventative measure against AD will only increase as early detection of AD is realized [27]. The SG inhibitors studied in this report have been developed using a rational drug design approach (shown in Figure 1), beginning with a molecular dynamics (MD) simulation screen, followed by single molecule biophysics experimental validation. In this report, we build on these studies with in vitro cell viability assays to further validate their neuroprotective role in HT22 cells. These new compounds, in conjunction with other Aβ targeted drugs such as mAbs, may be the way forward to prevent neurodegeneration caused by Aβ in AD.

### 1.2. SG Inhibitor Design Rationale

The synthetic pseudo-peptide inhibitors, designated SG, were designed and screened in silico by Dr. Arvi Rauk’s group using a computer aided drug design approach. The inhibitors, designated as SGA, SGB, SGC, or SGD, were designed to bind to the aggregation-prone region of Aβ, Aβ(16–23) or KLVFFAED, with high specificity and high affinity [28,29,30,31,32,33,34]. The peptide motif was selected as this region has been shown to have the high affinity and specificity for full length Aβ [14,15,20]. By binding as a β-sheet, the peptide can interfere with the aggregation of Aβ into neurotoxic oligomers. Propagation is inhibited by the placement of methyl groups on one side of the peptide backbone. The peptide is limited to eight residues to avoid it eliciting an immune response [32,33,34]. The peptide terminates in charged residues complementary to K16, E22, and D23 of Aβ, to improve binding affinity and direct the SG-Aβ complex into antiparallel (SGA, SGB) or parallel (SGC, SGD) β-sheets. Unnatural amino acid residues are incorporated to improve resistance to enzymatic degradation. These include D-residues (SGB, SGD) as well as L-residues (SGA, SGC). Selection of the specific residues was made on the basis of sophisticated docking using molecular operating environment (MOE), and the affinities assessed by MD simulations and umbrella sampling. Recognizing that BBB penetration may be a problem, SG inhibitors were myristoylated in the hope that it may be recognized by a fatty acid transporter in the BBB.

**Figure 2:**
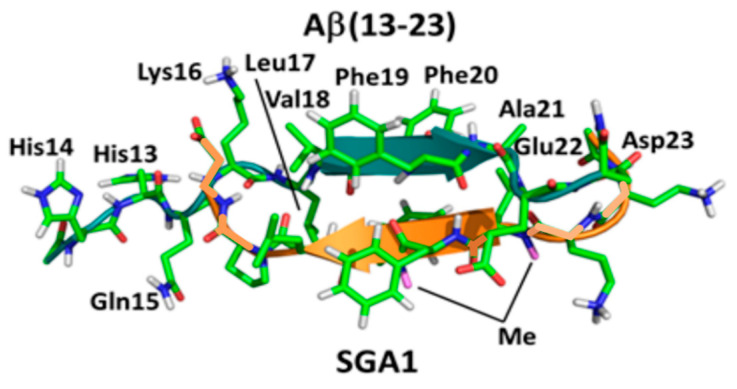
SG-Aβ(13–23) complex. Aβ backbone is shown in green, SGA1 is shown in orange. N-methylated peptide backbone (Me) appears on the bottom face of the complex.

Previous MD simulations predicted that SG inhibitors have a rigid backbone that orients with the N-methyl groups on the outer face of the SG-Aβ complex [28,30,31,33], Figure 2 shows the most probable predicted binding of an SG inhibitor with Aβ(13–23), which is designed to bind Aβ in antiparallel orientation. The N-methyl groups on the outer face are hypothesized to prevent the growth of β-sheet oligomers by blocking inter- and intra-molecular hydrogen bonding of Aβ peptides. In addition, MD simulations predict that SG inhibitors can have edge and site specificity to Aβ [31]. Since both faces of the Aβ peptide are available for aggregation effectively, inhibiting both the top and bottom faces would be important to completely inhibit aggregation. It is important to note that a high homodimer stability of the SG inhibitors would lower the effective net binding affinity to Aβ and needs to be considered for optimal target engagement [30]. Table 1 shows the previously calculated homodimer dissociation energy (ΔG_dimer_) and the average effective dissociation energy (ΔG_eff_) of several SG inhibitors tested in this paper. Moreover, ΔG_eff_ considers homodimer stability (ΔG_dimer_) and the average dissociation energy between the top and bottom face of Aβ. A negative ΔG_eff_ implies that SG-Aβ complexes are more stable than Aβ-Aβ complexes, for more information on how these values were calculated see [30]. Table 2 lists the amino acid sequences for the SG inhibitors studied in this report.

The substitution of proline residues to the basic SG inhibitor templates may disrupt β-sheet interactions, prevent aggregation, and even disassemble preformed Aβ fibrils [20,22,35]. Further modifications can improve peptide properties, for example: terminal charged amino acid residues can increase solubility [17]; synthetic, N-methylated and D-amino acid residues can improve both proteolytic stability and target engagement [16,19,25,34,35,36]; and the addition of shuttle peptides and hydrophobic residues could improve the blood-brain barrier (BBB) permeation [24,35]. There are major challenges associated with drug delivery to the brain; most importantly, the restriction presented by the BBB, which is known to exclude all but 2% of drugs [37]. The N-methyl backbone of the SG inhibitors may improve cell membrane translocation and BBB permeability by reducing the backbone electrostatic contributions and increasing the hydrophobicity of the peptide. To further improve BBB uptake as well as to increase localization near the membrane, the major site of Aβ aggregation, SG inhibitors with a myristic acid tail on the N-terminus have been proposed [33,38,39].

### 1.3. Target Verification by Single Molecule Force Spectroscopy

**Figure 3:**
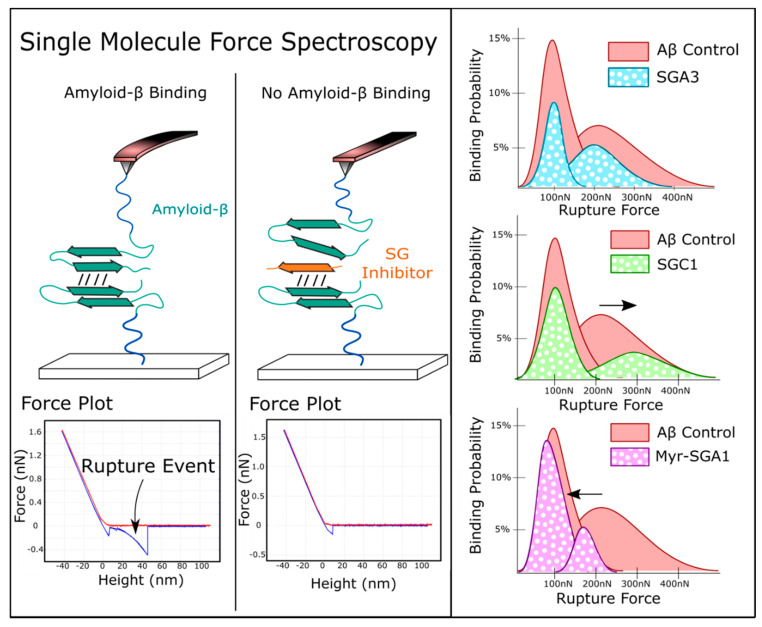
Illustration of previous. Single Molecule Force Spectroscopy experiments that showed Aβ(1–42)-Aβ(1–42) unbinding forces are modified by SG inhibitors. This suggests inhibitors block Aβ(1–42)-Aβ(1–42) binding orientations in unique fashion, results previously published in Mehrazma et al. 2017.

In Leonenko’s group, we previously demonstrated that these SG inhibitors effectively prevented Aβ dimerization using a single-molecule force spectroscopy (SMFS) biosensor approach (Figure 3) [21,30,40,41]. In a proof-of-concept study, we showed that SGA1 decreases the number Aβ binding events in a concentration dependent manner with increased effectiveness at higher concentration [21]. Later, the effects of other inhibitors (SGA3, SGC1 and Myr-SGA1) were compared, and it was confirmed that they all reduced the binding probability of Aβ, although no significant difference between inhibitors was found [30]. In further analysis of the unbinding force distribution, we found that inhibitors shifted the Aβ binding probability distribution in unique ways. SGC1 increased the binding probability at higher force, Myr-SG1 decreased the binding probability at higher force, and SGA3 more uniformly reduced binding probability across the unbinding force distribution, shown in Figure 3 below [30]. This may suggest that the SG inhibitors have different likelihoods of blocking different binding configurations of Aβ; for instance, they may preferentially block parallel/anti-parallel binding orientations, or may block different types of intermolecular forces, such as hydrophobic interactions, electrostatic or hydrogen bonding [30].

### 1.4. Structural Characterization of SG-Aβ Aggregation

Experimental validation of the effect of SGA1 on amyloid aggregation and structure was performed previously by several methods: including ThT fluorescence, circular dichroism (CD), Western blot and AFM imaging. The influence of SGA1 on Aβ(1–40) and Aβ(1–42) aggregation by ThT fluorescence assay was performed at a concentration of 25 μM Aβ(1–40) and Aβ(1–42) at 37 °C for 5 to 7 days, across a broad range of SG inhibitor concentration to determine the IC_50_ inhibitory ligand:Aβ ratio [33]. For SGA1, the IC_50_ ligand:Aβ(1–40) ratio occurred at 0.2, while the control, N-methylated KLVFF peptide (Ac-K(me)LV(me)FF-NH2), was approximately 2.0 [33]. The IC_50_ inhibitory ligand/Aβ1–42 ratio was approximately double for SGA1 of 0.5 compared to the control N-methylated KLVFF peptide 4.0 [33]. The results from ThT fluoresence assay are further supported by Western blot analysis, which demonstrated similar stoichiometric molar ratios of SG inhibitor to Aβ(1–42) resulted in the complete inhibition of higher molecular weight oligomers and reduction in dimers and trimers in a concentration-dependent manner [33].

**Figure 4:**
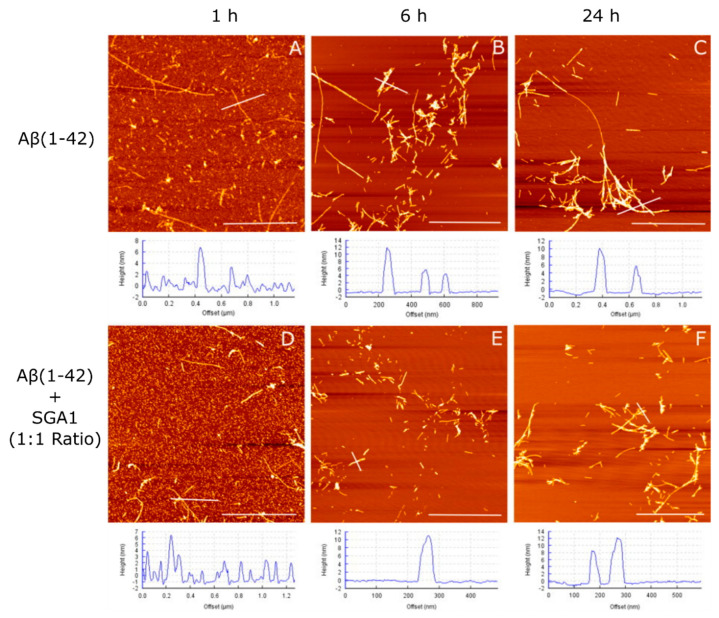
AFM images of Aβ(1–42) with and without SGA1 on freshly cleaved mica. Control Aβ(1–42) at 110 µM after (**A**) 1 h, (**B**) 6 h, and (**C**) 24 h and with equimolar concentration of SGA1 after (**D**) 1 h, (**E**) 6 h and (**F**) 24 h, adapted with permission from Hane et al., Biosensors and Bioelectronics; published by Elsevier, 2013. [21].

AFM imaging studies of Aβ aggregation were carried out independently by the Leonenko lab that demonstrates the anti-aggregation properties of the SGA1 inhibitor [21]. In Hane et al., solutions of Aβ(1–42) (prepared by the Fezoui method) with and without SGA1 were deposited onto freshly cleaved mica at 1:1 ratio with a final concetration of 110 μM Aβ(1–42), and then incubated for 1, 6 and 24 h, before being washed, dried and than imaged by AFM (Figure 4, adapted with permission) [21]. Fibril length of Aβ(1–42) aggregates was greatly reduced in the presence of the SG inhibitor compared to the Aβ-only control [21]. In addition, the amount of fibrils and oligomers were quantified across the time points, showing that SG inhibitor decreased the amount of Aβ(1–42) fibrils compared to oligomers [21]. In paying closer attention to the 1 h timepoint, the surface roughness of the background for Aβ(1–42) without inhibitor suggesting that there is a higher degree of absorption of the amyloid aggregates on the surface compared to Aβ(1–42) in the presence of SG inhibitors. At 1 h, when SG inhibitor is present, mica background can be seen below the amyloid aggregates, while for the Aβ(1–42) control, it appears as though a thicker layer of smaller aggregates is present [21]. This may suggest that the SG inibitor when complexed with Aβ may block the ability of Aβ(1–42) aggregates from absorbing onto the surface of the mica by preventing hydrogen bonding between Aβ(1–42) and the mica surface, and are thus more easily washed away during sample preparation.

### 1.5. Cell Viability Studies.

**Figure 5:**
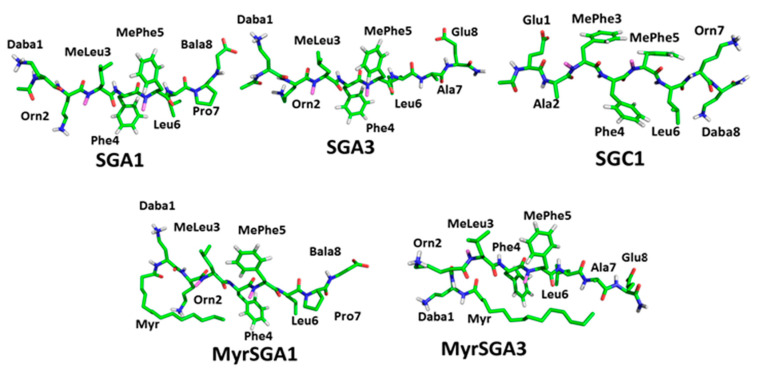
Structure of SG inhibitors tested in the drug toxicity and Aβ(1–42) rescue experiments presented in this report.

In this report, we extend our previous computational and biophysical studies to test five SG inhibitors in cell viability studies, which were done in the Beazely laboratory. The five inhibitors are shown below in Figure 5. We demonstrate that two of the SG inhibitors, SGA1 and SGA3, improve the cell viability of the mouse neuronal cell line HT22 in Aβ rescue experiments compared to Aβ(1–42) oligomers alone. We also discovered that both myristoylated inhibitors were intrinsically toxic to cells and further potentiated the toxicity of Aβ oligomers. We compare these cell viability studies in the discussion section to previous biophysical studies and synthesize our knowledge learned across the three different methodological approaches: molecular dynamics simulations, single molecule biophysical studies, and in vitro cell viability assays.

## 2. Results

### 2.1. SG Inhibitor Toxicity

**Figure 6:**
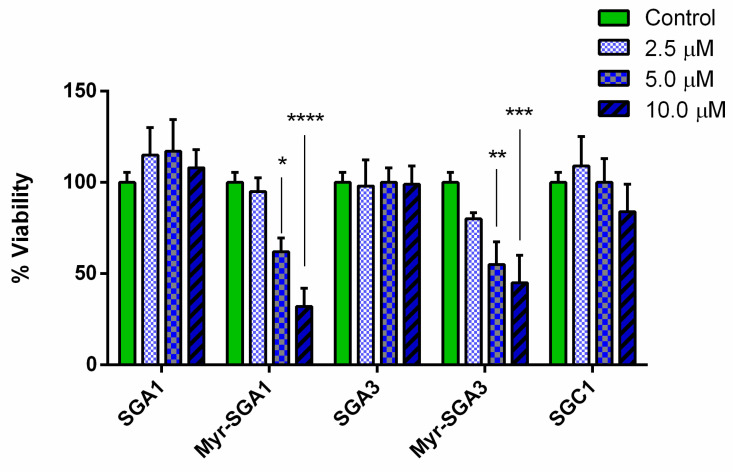
HT22 cells treated with SG inhibitors for 24 h. Cell viability was assessed by MTT assay and expressed as a percentage of untreated control, error bars represent SEM, *n* = 4 for each concentration and inhibitor, * *p* < 0.05, ** *p* < 0.01 *** *p* < 0.005, and **** *p*< 0.001, one-way ANOVA (α = 0.05) with Dunnett’s multiple comparison test was performed to assess statistical significance.

Before evaluating the potential protective effects of the compounds against Aβ(1–42) toxicity, we determined whether these compounds exhibited any toxicity in vitro (Figure 6). We found that SG inhibitors are largely non-toxic up to 10 µM, except for the myristic acid-modified compounds. Myristic acid-modified inhibitors demonstrated a dose-dependent toxicity for both Myr-SGA1 and Myr-SGA3, with significant reductions in HT22 cell viability at 5 and 10 µM. The IC_50_ for the toxicity of the Myr-SGA1 and Myr-SGA3 were both approximately 7.5 µM, whereas for standard inhibitors, no significant reduction in cell viability was observed over the range of concentrations tested here, therefore IC50 could not be calculated for SGA1, SGA3 and SGC1.

### 2.2. The Effects of SG Inhibitors on Aβ Oligomer Toxicity

**Figure 7:**
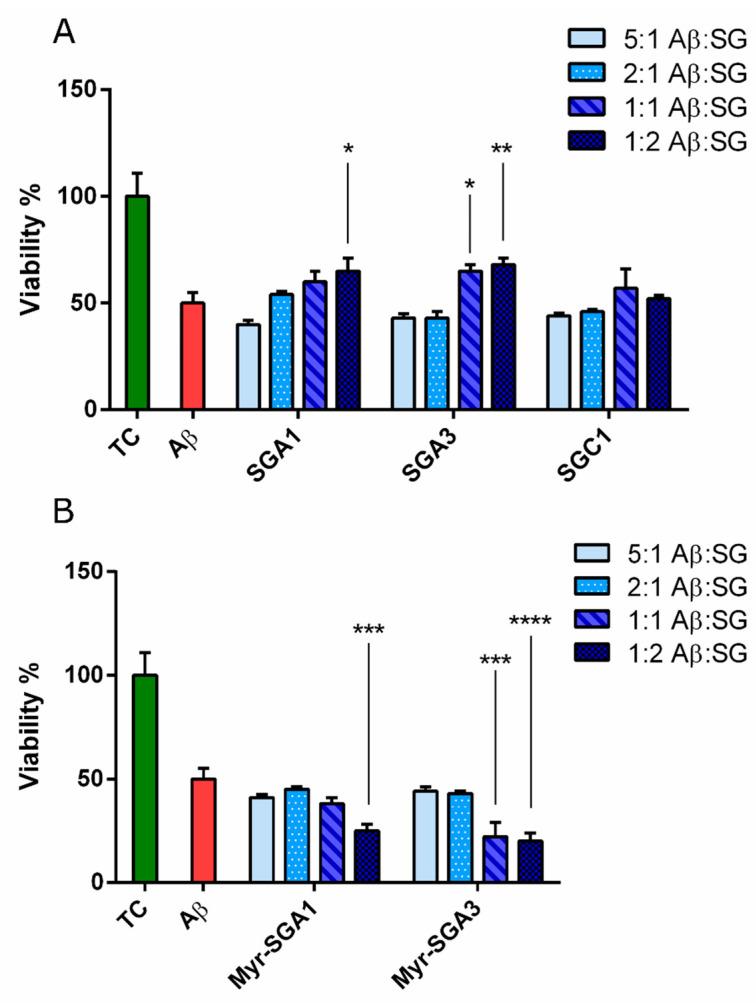
Effect of SG Inhibitors on Aβ(1–42) Toxicity. Mean (with SEM) of HT22 cell viability as measured by MTT assay and expressed as a percentage of untreated control. Cells were treated with Aβ (*n* = 6) or Aβ with various ratios of SG inhibitor 5:1 and 2:1 and 1:1 and 1:2 (*n* = 3) of Aβ/SG, for 24 h. (**A)** Standard inhibitors and (**B)** Myristoylated SG inhibitors (bottom). Significance was assessed by one-way ANOVA (α = 0.05) performed with Dunnett’s multiple comparison to Aβ-treated group: * *p* < 0.05, ** *p* < 0.01, *** *p* < 0.005, and **** *p* < 0.001.

The protective effects of SG inhibitors on Aβ(1–42) oligomer toxicity, as assessed in the HT22 cell model, were small but promising (Figure 7A) when considering the protective effects from Aβ(1–42) observed in similar studies in HT22 cells [42]. Aβ(1–42) oligomers (5 µM) caused a reduction in viability to 50 ± 5% of true control; this is in line with other in vitro assays in SHSY5Y cells and HT22 cells which typically observe between 50–60% reduction in cell viability at this concentration [42,43]. Two inhibitors (SGA1* and SGA3**) improved the cell viability of HT22 cells to 65 ± 6% and 68 ± 3% at twice the molar concentration of Aβ (*p* < 0.05 * and *p* < 0.01 **, respectively), meanwhile SGA3 caused an improvement in cell viability at equimolar concentration of Aβ (*p* < 0.05 *) to 64 ± 3% of true control. SGC1 showed no statistically significant effect on Aβ(1–42) toxicity at the range of concentrations tested here. The toxicity of Myr-SGA1 and Myr-SGA3 inhibitors appeared to compound with that of Aβ(1–42), reducing cell viability to below 30% (Figure 7B). There was a trend toward increased toxicity at low concentration, though compared to Aβ(1–42) treated control the effect was not statistically significant. DMSO used as vehicle (at 0.1%) also caused small reduction in HT22 cell viability, but was also not statistically significant (data not shown).

**Figure 8:**
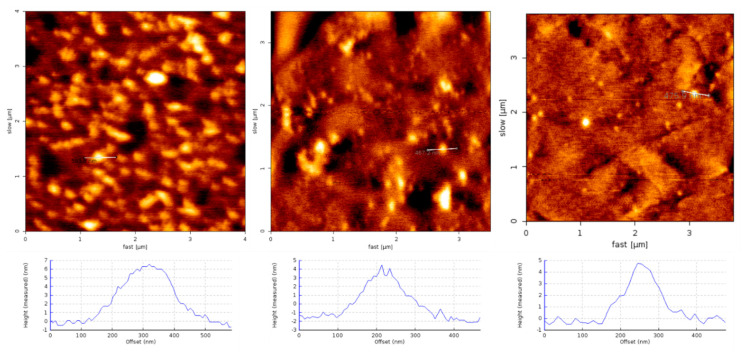
AFM images of Aβ(1–42) oligomers prepared at 5 µM for 24 h at 4 °C in DMEM/F12 media deposited onto freshly cleaved mica, left panel Aβ(1–42) control, middle panel 1:1 SGA1 to Aβ(1–42) and right panel 2:1 SGA1 to Aβ(1–42).

AFM was used to confirm the structure of 5 μM Aβ(1–42) and 5 μM Aβ(1–42) with SG inhibitor prepared in DMEM/F12 media similar to our previous report using the Stine protocol [43,44]. The 5 μM Aβ(1–42) and 5 μM Aβ(1–42) with SG inhibitor solutions prepared in DMEM/F12 media were incubated for 30–60 min on freshly cleaved mica followed by washing and drying. Representative images of oligomers, approximately 2 to 8 nm, were produced and no fibrils were detected for any of the control or SG inhibitor Aβ(1–42) solutions (Figure 8). Moreover, incubation at 5 μM Aβ(1–42), compared to 100 μM in the unmodified Stine protocol, should slow aggregation rates favoring low molecular weight and intermediate oligomeric Aβ(1–42), rather than fibrillar Aβ(1–42). Heterogeneous background was observed due to other media components absorbing onto the mica, forming a thin film.

## 3. Discussion

### 3.1. In vitro HT22 Cell Models for Testing Anti-Aβ Aggregation Drugs

The murine hippocampal-derived cell line HT22 was used in this study to assess cell viability to Aβ(1–42) insult and ability of SG inhibitors to protect against this insult. HT22 cells are sensitive to glutamate excitotoxicity [45], express neuronal cholinergic markers [46]. These characteristics make HT22 cells suitable for evaluating the effects of Aβ on cell viability, as cholinergic dysfunction and glutamate excitotoxicity are expected to be involved in Aβ pathology and AD [47,48]. Most importantly, HT22 cells are sensitive to Aβ toxicity, as previous studies have examined the ability of neurotransmitters or hormones to bolster cell viability after Aβ exposure [42,49]. These treatments will likely increase cell proliferation and metabolic activity through receptor signaling pathways, in addition to any effects of these small molecules on aggregation-dependent toxicity. For instance, treatment of nicotine at 10 μM, 100 μM and 500 μM improved cell viability of HT22 cells treated with 5 μM Aβ(1–42) by approximately 10% to 20% [42]. This is comparable to the 15–18% improvement in cell viability we observed here with SGA inhibitors at 5–10 μM.

The trend towards decreased viability at low concentration was not statistically significant. In principle, if inhibitors slowed the fibrillization process, it may stabilize toxic Aβ(1–42) species. Considering previous ThT studies of SGA1, the IC_50_ inhibitory ratio was approximately 5:2 Aβ/SG ratio, which is between the lowest two concentrations we tested here. Thus, we expect that we are not at complete Aβ inhibition at low concentration. In addition, based on AFM imaging studies at 1:1 Aβ/SG ratio, we observed an increased oligomer/fibril ratio, suggesting that at higher Aβ, even at equimolar SG concentration, complete inhibition is not possible, though some protection is afforded. 

This was the first report of the effects of Aβ aggregation inhibitors in HT22 cells; the MTT cell viability results reported here verify the sensitivity of HT22 cells to 5 µM Aβ(1–42) oligomers. There is a complex equilibrium between the inhibitor and amyloid complexes (in this case: SG-SG, SG-Aβ and Aβ-Aβ) in solution. Inhibitors are expected to be most effective when amyloid concentrations are low. However, to achieve an appreciable toxic effect over short-time scales, in vitro super-physiological concentrations of Aβ must be used. Thus, caution in predicting the efficacy of amyloid inhibitors in vivo from in vitro assays is warranted. Inherent in the strategy of Aβ aggregation is relying on physiological clearance mechanisms to remove monomeric species before they can aggregate, these would include BBB efflux and proteolytic breakdown and recycling. In acute treatment, in vitro assays do not accurately reflect this crucial portion of the mechanism, thus the assay is working against the intervention, which may explain the modest increase in neuroprotection that we observed. Overall, this style of acute assay is likely more suited for studying signaling and metabolic interventions rather than aggregation inhibitors, though this assay is still quite useful to rule out toxic compounds and identify potential candidates for further study.

### 3.2. Myristic Acid to Improve BBB Delivery of Peptides

Most central nervous system drugs are not able to permeate through the blood-brain barrier, especially drugs with a molecular weight greater than 500 Daltons [37]. As the SG inhibitors are greater than 500 Da, they will likely require a mechanism to facilitate transport across the BBB [37], thus the myristoylation of the SG inhibitors was proposed. This may improve passive diffusion by increasing hydrophobicity of the peptide, or via receptor-mediated translocation of fatty acid transporters [37]. The lipidation of proteins is a common post-translational modification that can localize the protein near the membrane and result in direct binding, insertion, and trafficking into the lipid bilayer [50,51]. Myristoylation of the SG inhibitors could localize the inhibitor in or near the membrane environment where Aβ toxicity is initiated [52,53]. Interestingly, the myristoylation of the SG inhibitors resulted in direct cytotoxicity that was not observed in non-lipidated inhibitors. Saturated fatty acids (palmitic and stearic acids) have been shown to induce AD-like tau hyperphosphorylation, as well as trigger caspase-dependent and independent cell death mechanisms [54,55]. Though the peptide inhibitors are not strictly speaking free fatty acids, they may be regarded as fatty acid derivatives. The molecular mechanisms of lipotoxicity are expected to include ER, mitochondrial and oxidative stress [56]. Lipotoxicity has also been attributed to detergent-like and destabilizing effects on cellular membranes [56]. It is possible that myristoylated inhibitors are solubilizing or permeabilizing the outer cell and intracellular membranes. In addition, MD simulations show that Myr-SG inhibitors have a high homodimer stability, suggesting that they themselves may aggregate, leading to toxicity [30]. Finally, the enhanced cytotoxicity of the Myr-SG inhibitors may be due to their ability to insert themselves into the lipid bilayer and act as a seed for oligomerization, which is expected to be involved in the earliest stages of Aβ toxicity. Our study demonstrates that myristoylation may not be a suitable strategy to increase the transport of peptide aggregation inhibitor drugs across the BBB.

### 3.3. Comparing SG Inhibitors In Silico, Single Molecule Force Spectroscopy and Cell Viability Assays

The inhibitors tested in this study were designed through computer-aided in silico drug design. Ligand-docking protocols and steered MD simulations were used to calculate a predicted affinity between the SG inhibitor and the target Aβ(13–23), the self-recognition region (R) of the peptide HHQKLVFFAED [33]. In the computational studies, the self-recognition region, Aβ(13–23), is expected to be very flexible, with a hairpin turn between V18 and F19, forming an intramolecular β-sheet; this hairpin turn is stabilized by intramolecular interactions between the carboxylate group of E22 and the backbone N-H bonds of V18, F19 and F20 [28,33,57]. Computational studies demonstrated that the SG inhibitors have a rigid backbone in comparison to Aβ(13–23), due to the N-methylated amine backbone which cannot form intramolecular hydrogen bonds and that the SG-R complex prevents the hairpin turn of Aβ(13–23) increasing rigidity (Figure 2) [21,28,30,31]. Various substitutions of the SG inhibitor are made to maximize affinity for Aβ13–23. N-terminal substitution of γ-diaminobutyric acid may improve interactions with D23 on Aβ; substitution of lysine in the KLVFF sequence with ornithine may improve electrostatic side chain interactions with E22 [33,57]. Other improvements to peptide inhibitors can be made by substituting various lipophilic aromatic residues, which may optimize hydrophobic interactions between the drug and Aβ target [58,59].

Here, we demonstrate that SGA inhibitors, that are predicted to bind in an antiparallel fashion, have a significant protective effect against Aβ(1–42) toxicity. In contrast, SGC1, predicted to bind in parallel fashion, had no effect on toxicity (Figure 3). In addition to the preferred binding orientation (parallel or anti-parallel), MD simulations have revealed that SG inhibitors can have preferred site and edge specificity to the top (RT) and bottom (RB) of Aβ(13–23), and presumably full length Aβ; therefore, there are four possible binding orientations of the SG-Aβ complex. To efficiently inhibit oligomerization, both top and bottom sites should be blocked by the inhibitor. In addition to the preferred binding configuration, inhibitors have some homodimer affinity (SG-SG complex) [21,30], which must be minimized to prevent competition for target engagement with Aβ [31]. MD simulations and affinity calculations previously revealed that SGA1 is predicted to have preferential specificity for RT over RB, as indicated by the higher dissociation energy to RT and had relatively low homodimer dissociation energy [31]. SGA3 had high dissociation energies to both RT and RB, but also had a high homodimer stability compared to SGA1 [31]. This may explain the increase in protection that SGA3 afforded when compared to SGA1 at equimolar concentrations of Aβ(1–42). When considering the edge and site specificity for SGC1, the computational study predicted that SGC1 had the most favorable predicted binding characteristics compared to SGA1 and SGA3, low homodimer stability and high Aβ binding. However, SGC1 did not have any effect on Aβ(1–42) toxicity, suggesting that a parallel binding orientation is much less protective than an anti-parallel orientation, despite more favorable effective ΔG. The SG inhibitors tested here are L-amino acid inhibitors that are more prone to degradation by endogenous proteases than the corresponding D-amino acid peptides, so we expect that the SGB and SGD compounds would be more effective. Moreover, the D-amino acid analogs appear to have more favorable binding characteristics [29].

In addition to the MD simulations, we previously conducted an SMFS biophysical study to test ability of SGA3, SGC1 and Myr-SGA1 to partially inhibit full length Aβ(1–42)-Aβ(1–42) dimerization demonstrating unique inhibiting properties of each inhibitor [30]. In these experiments, direct measurements of Aβ dimerization were made, wherein the unbinding force of Aβ was measured with and without SG inhibitor. In general, there was a bimodal distribution of rupture forces; this distribution could be attributed to two non-mutually exclusive mechanisms: first, that lower energy binding forces may correspond to hydrophobic interactions and higher energy rupture forces to the formation of hydrogen bonds; and second, to parallel and anti-parallel binding orientations. All inhibitors reduced the probability of binding, with no overall differences in the total number of events, however there were differences in the resulting force distributions, suggesting unique SG-Aβ(1–42) complex formation. SGC1 increased the most probable unbinding force of the second mode of the distribution, this may be due to blocking parallel (lower energy) rather than antiparallel (higher energy) binding configurations, which could help to explain the limited effectiveness at protecting HT22 cells from Aβ(1–42) toxicity [30]. SGA3 evenly reduced binding events across the histogram. In looking at Myr-SGA1 in SMFS experiments, we see a dramatic ability of the inhibitor to completely inhibit higher energy binding configurations but inability to prevent any of the lower energy binding configurations. This may indicate that Myr-SGA1 blocks hydrogen bonding (which could correspond to the higher energy state), but is unable to block lower energy hydrophobic interactions due to the myristic acid tail, thus the Myr-SG/Aβ could be stabilizing the more cytotoxic forms of Aβ oligomers.

## 4. Materials and Methods

### 4.1. Reagents and SG Inhibitors

First, 1,1,1,3,3,3-hexafluoro-2-propanol (HFIP) (>99%), DMEM/F12 media with L-Glutamine, Fetal Bovine Serum (FBS), Penicillin/Streptamycin (Pen/Strep), Dimethylsulfoxide DMSO (>99%), 3-(4,5-dimethylthiazol-2-yl)-2,5-diphenyltetrazolium bromide (MTT), Triton X-100. Amyloid-β, >97% pure, HFIP purchased from rPeptide (Bogart, GA, USA). SG inhibitors (>95% pure) were synthesized by AnaSpec Inc. (Fremont, CA, USA); the sequences are shown in Table 2.

### 4.2. HT22 Cells and MTT Assay

The MTT assay was used to evaluate the viability of HT22 cells exposed to inhibitor or mixtures of inhibitor with full length Aβ(1–42). HT22 cells were plated into 96-well cell culture plates at a cell density of 100,000 cells/mL in full growth media (DMEM/F12, with 10% FBS, 1% Pen/Strep) at 37°C, 5% CO2, until 80% confluence (20–22 h). Full growth media was exchanged for treatment media (containing Aβ or Aβ with SG inhibitor). After treatment, cells are then returned to incubator for 24 h. The media was then exchanged for phenol red-free DMEM/F12 containing 0.5 mg/mL MTT. Cells were returned to the incubator to metabolize the MTT for 3.5 h, after which, cells were solubilized in isopropanol, with 10% Triton X-100 and 0.1 M HCl. After solubilization, absorbance from the 96-well plates was read in Molecular Dynamics™ plate reader at 570 nm and 690 nm. The signal at 690 nm and media control was subtracted from the 570 nm reading prior to analysis. HT22 cells were a generous gift from Dr. Robert Cumming, PhD, University of Western Ontario. 

### 4.3. Aβ and Aβ-SG Inhibitor Treatment Preparation and AFM Characterization

Aβ(1–42) oligomers were prepared following protocols adapted from Stine et al. [44]. Briefly, Aβ(1–42) was dissolved in HFIP to 1 mg/mL solution and aliquoted into microcentrifuge tubes, and allowed to evaporate under desiccant for 24 h, leaving behind a thin film of Aβ(1–42) monomers, which was stored at 2-0 °C under desiccant. Immediately prior to use, Aβ(1–42) monomers were suspended in dimethyl sulfoxide (DMSO) at a concentration of 5 mM, vortexed for 30 s, pulse centrifuged for 30 s and then sonicated for 10 min at room temperature. The 5 mM Aβ(1–42) monomer solution prepared in DMSO was then diluted to 100 µM in cold DMEM/F12 media, then further diluted to 5 µM in fresh DMEM/F12 or DMEM/F12 containing SG inhibitor at various concentrations corresponding to a final ratio of 5:1, 2:1, 1:1 and 1:2 Aβ to SG. Solutions of 5 µM Aβ(1–42) (as control), or 5 µM Aβ(1–42) with SG inhibitor were incubated at 4°C for 24 h under conditions favorable to produce intermediate oligomeric and smaller Aβ(1–42) species.

Aβ(1–42) oligomer species with and without SG inhibitor were confirmed by AFM imaging. Briefly, the 5 μM Aβ(1–42) and 5 μM Aβ(1–42) with SG inhibitor solutions (various ratios) in DMEM/F12 media were incubated for 30–60 min on freshly cleaved mica, followed by three washes with ultrapure water (MilliQ). Mica slides were dried using nitrogen and imaged with NCST soft tapping mode cantilevers (Nanoworld, Neuchâtel, Switzerland) using intermittent contact mode on the JPK Nanowizard 2 (JPK, Berlin, Germany).

### 4.4. Statistical Analysis

Inhibitor toxicity to HT22 cells was assessed by treating cells with inhibitor (SGA1, Myr-SGA1, SGA3, Myr-SGA3 or SGC1) at concentrations of 2.5, 5.0 and 10.0 µM for 24 h in triplicates, for *n* = 4 repeats. One-way ANOVA with Dunnett’s multiple comparisons test was performed to establish significance between inhibitor groups and vehicle-treated control, threshold for significance: α = 0.05. To assess SG inhibitor neuroprotection against Aβ(1–42) oligomers, Aβ(1–42) alone or Aβ(1–42) mixed with each inhibitor at the ratios described above were applied to cells in quadruplicates for *n* = 3 repeats. One-way ANOVA with Dunnett’s multiple comparisons was performed to establish significance between Aβ-SG inhibitor mixture and Aβ-treated control; threshold for significance: α = 0.05.

## 5. Conclusions

We showed that several SG inhibitors demonstrated promising ability to protect HT22 cells from Aβ(1–42) toxicity. Based on previous studies, the mechanisms involve binding to Aβ(1–42) and preventing its misfolding, and more specifically that the anti-parallel (SGA) binding inhibitors are more likely to protect cells than those that bind in a parallel (SGC) orientation. We also showed that myristic acid modified SG-peptides are neurotoxic themselves and may not be suitable as amyloid prevention drug candidates. We suggest that this in vitro study justifies further screening of a larger set of SG inhibitors in more physiologically relevant in vivo models. As this is the first such in vitro test of anti-Aβ aggregation compounds in HT22 cells, it sets a benchmark for future in vitro studies of amyloid aggregation inhibitors in this cell line. Testing of SGB inhibitors, the D-enantiomers of the anti-parallel binding SG inhibitors is the next step in the drug development pipeline.

## Figures and Tables

**Table 1:** SG Inhibitor classes and predicted binding characteristics of select SG inhibitors tested in this report. SGA and SGC inhibitors were tested in this report.

**SG Inhibitor Classes**	**Anti-Parallel**	**Parallel**
L-enantiomer	SGA	SGC
D-enantiomer	SGB	SGD
**SG Inhibitor Binding**	**ΔG_dimer_ (kJ/mol)**	**Average ΔG_eff_ (kJ/mol)**
SGA1	21	3
SGA3	46	-4
SGC1	26	-24
Myr-SGA1	62	6

**Table 2:** The sequences of SG inhibitor pseudo-peptides. Legend: β—alanine (Bala), N—methylated backbone residue (Me), diaminobutyric acid (Daba), ornithine (Orn), and myristic acid tail (Myr).

SG Inhibitor	Inhibitor Sequence
SGA1	Daba-Orn-(Me)Leu-Phe-(Me)Phe-Leu-Pro-Bala
MyrSGA1	Myr-Daba-Orn-(Me)Leu-Phe-(Me)Phe-Leu-Pro-Bala
SGA3	Daba-Orn-(Me)Leu-Phe-(Me)Phe-Leu-Ala-Glu
MyrSGA3	Myr-Daba-Orn-(Me)Leu-Phe-(Me)Phe-Leu-Ala-Glu
SGC1	Glu-Ala-(Me)Phe-Phe-(Me)Phe-Leu-Orn-Daba

## Data Availability

The data presented in this study are available in the article.

## Abbreviations

Aβ	amyloid-β
AD	Alzheimer’s disease
ANOVA	analysis of variance
BBB	blood-brain barrier
CNS	central nervous system
DMSO	dimethylsulfoxide
HFIP	hexafluoroisopropanol
MD	molecular dynamics
MOE	Molecular Environment
MTT	3-(4,5-Dimethylthiazol-2-yl)-2,5-Diphenyltetrazolium Bromide
mAb	monoclonal antibody
SMFS	single-molecule force spectroscopy
